# The impact of lifetime interpersonal and intentional trauma on cognition and vulnerability to psychosis in bipolar disorder

**DOI:** 10.1192/bjo.2021.991

**Published:** 2021-09-09

**Authors:** Julia G. Lebovitz, Caitlin E. Millett, Meg Shanahan, Nomi C. Levy-Carrick, Katherine E. Burdick

**Affiliations:** Mood and Psychosis Research Program, Department of Psychiatry, Brigham and Women's Hospital, MA, USA; and Department of Psychiatry, Harvard Medical School, MA, USA; Mood and Psychosis Research Program, Department of Psychiatry, Brigham and Women's Hospital, MA, USA; and Department of Psychiatry, Harvard Medical School, MA, USA; Mood and Psychosis Research Program, Department of Psychiatry, Brigham and Women's Hospital, MA, USA; Mood and Psychosis Research Program, Department of Psychiatry, Brigham and Women's Hospital, MA, USA; and Department of Psychiatry, Harvard Medical School, MA, USA; Mood and Psychosis Research Program, Department of Psychiatry, Brigham and Women's Hospital, MA, USA; and Department of Psychiatry, Harvard Medical School, MA, USA

**Keywords:** Psychosis, trauma, bipolar affective disorders, gender differences, cognition

## Abstract

**Background:**

Studies have shown that over half of individuals with bipolar disorder experience early-life trauma, which may influence clinical outcomes, including suicidality and presence of psychotic features. However, studies report inconsistent findings regarding the effect of trauma on cognitive outcomes in bipolar disorder.

**Aims:**

Our study explores the effect of lifetime trauma on the level of vulnerability to psychosis and cognitive performance in participants with bipolar disorder.

**Method:**

We evaluated lifetime trauma history in 236 participants with a diagnosis of bipolar disorder type 1 or 2, using the Structured Clinical Interview for DSM-IV and the Childhood Trauma Questionnaire. We classified trauma types based on the Substance Abuse and Mental Health Services Administration's concept of trauma, which characterises the type of experienced trauma (e.g. interpersonal and intentional, accidental or naturally occurring). Our primary outcome measures of interest were vulnerability to psychosis (Schizotypal Personality Questionnaire), cognitive performance (MATRICS Consensus Cognitive Battery) and social functioning (Social Adjustment Scale Self-Report).

**Results:**

Multivariate analysis of covariance showed a significant effect of trauma type on the Schizotypal Personality Questionnaire cognitive–perceptual domain (F(3) = 6.7, *P* < 0.001). The no-trauma group had lower cognitive–perceptual schizotypal features compared with the accidental and intentional trauma (*P* < 0.001) and interpersonal and intentional trauma (*P* = 0.01) groups.

**Conclusions:**

Our results highlight the need for careful trauma inquiry in patients with bipolar disorder, and consideration of how trauma-focused or -informed treatments may be an integral part of treatment planning to improve outcomes in bipolar disorder.

## Bipolar and trauma overview

Bipolar disorder is a mood disorder characterised by affective dysregulation and impaired cognition.^1^ It is commonly reported that approximately half of patients with bipolar disorder experience childhood trauma.^2^ Studies have found that interpersonal traumas, such as physical abuse and sexual assault, are more prevalent in participants with severe mental illness compared with the general population.^3^ Patients with bipolar disorder who have a history of trauma are at increased risk for a range of poor clinical outcomes,^4^ including an elevated risk for suicide attempts, more frequent diagnosis of rapid cycling^2^ and an increased rate of psychosis and psychotic-like traits.^5^

Traumatic childhood experiences have been related to schizotypal personality characteristics, particularly suspiciousness, in healthy controls as well as participants diagnosed with bipolar disorder and schizophrenia.^6^ In individuals with schizotypal personality disorder, emotional neglect is associated with pathological interpersonal traits (excessive social anxiety and constricted affect), whereas sexual assault is correlated with high cognitive–perceptual load (ideas of reference, odd beliefs, magical thinking).^7^ There is evidence of a link between childhood traumatic experiences and delayed treatment initiation and impaired social functioning in adulthood in patients with an axis I psychotic disorder.^8^

There also appears to be a cumulative effect of trauma on several clinical outcomes in patients with bipolar disorder. One study found that compared with participants with bipolar disorder who reported no trauma, those who reported seven or more adverse childhood experiences (ACEs) were five times more likely to report hallucinations as part of their illness.^9^ Further, exposure to multiple ACEs has been correlated with earlier hospital admission, number of recent suicide attempts, revictimization in adulthood, health-risk behaviours, comorbidity of substance use, health problems and homelessness.^10^

There is also evidence supporting a gender-specific response to trauma in psychiatric populations. A study by Garcia et al found that, in people with psychotic disorder, there was a strong association between childhood trauma and increased psychotic symptoms, depressive symptoms and poorer functioning in females, but not in males.^11^ Following trauma exposure, females are more likely to develop an affective disorder and males are more likely to present with substance use.^12^ A review found gender-specific changes in brain structure and function following trauma exposure, notably loss of grey matter in the limbic system for males and overactivity in the amygdala for females, creating disruption in emotional processing networks.^13^

There are inconsistent findings as to the effect of trauma on cognition in bipolar disorder. Generally, early-life trauma has been correlated with impaired executive functioning in healthy adults,^14^ as well as impaired moral decision making,^15^ verbal intelligence^16^ and working memory^17^ for people with bipolar disorder. One systematic review showed an association between increased childhood trauma experience and poorer cognitive performance,^18^ whereas another systematic review found a small effect size between cognition and childhood trauma.^19^ One study found social cognitive deficits in people with bipolar disorder who were exposed to childhood trauma.^6^ Research also suggests the presence of gender-specific differences in cognitive performance based on trauma history. Our prior study, which used an affective processing task as the primary outcome, found that male participants with bipolar disorder who were exposed to emotional abuse in childhood had a response bias away from negative stimuli, whereas female participants with bipolar disorder with similar histories showed response biases toward negative stimuli.^20^

## Study objectives

However, very few studies exist that assess the cumulative burden of lifetime (childhood and adulthood) trauma on psychotic features and cognitive performance in bipolar disorder. The present study aims to fill this gap by using structured clinical interviews to assess lifetime history of trauma and its effect on clinical characteristics, vulnerability to psychosis, cognitive ability and social functioning in patients with bipolar disorder, and the gender-specific differences that exist therein. We aim to expand existing literature by categorising the trauma types based on the Substance Abuse and Mental Health Services Administration (SAMHSA) trauma-informed care models. Examining the type of trauma (intentional and interpersonal, accidental or natural) could help explain inter-group discrepancies. For example, in-patients with bipolar disorder who reported assaultive traumas were more symptomatic than patients that reported other types of traumas; interestingly, this study found that 40% of participants with bipolar disorder with psychotic features had been exposed to an assaultive trauma.^4^ Accidental traumas may elicit anger and frustration, whereas intentional and interpersonal traumas have been found to result in more shame and confusion.^21^

The goals of this present study were to assess the prevalence of specific trauma types in stable out-patients with bipolar disorder; the effect of trauma type on vulnerability to psychosis and cognition; the effect of cumulative burden of trauma on clinical features, vulnerability to psychosis, cognition and social function; and the role that biological gender plays in these relationships. Unlike most previous research, this study includes lifetime trauma as opposed to just childhood trauma, to understand how trauma may affect both the onset and clinical course of bipolar disorder.

## Method

### Participants

Data was extracted from a total of 264 participants recruited from the Icahn School of Medicine at Mount Sinai (ISMMS) and the Brigham and Women's Hospital (BWH) between 2012 and 2018. The authors assert that all procedures contributing to this work comply with the ethical standards of the relevant national and institutional committees on human experimentation and with the Helsinki Declaration of 1975, as revised in 2008. All procedures involving human patients were approved by institutional review boards [BWH approval number 2017P001269 and ISMMS approval number 13-00561]. All participants provided informed consent before the initiation of study procedures. Participants assessed at BWH were recruited via advertisements or through the Research Patient Data Registry. Participants recruited through the registry were called by a research staff and asked to participate. Participants from ISMMS were recruited through community advertisements. Participants that had incomplete data relevant to the main analyses (schizotypy, cognition) were excluded from the present study**.** Participants were diagnosed with bipolar disorder by the Structured Clinical Interview for DSM-IV (SCID-IV);^22^ aged 18–65 years old; stable at the time of testing (as indexed by a score of ≤3 (mildly ill) on the Clinical Global Impression for Bipolar Disorder (CGI-BD)^23^); and had no history of central nervous system trauma, neurological disorder, attention-deficit hyperactivity disorder, recent substance misuse/dependence (past 3 months) or history of electroconvulsive therapy in the past year. Some patients had mild-to-moderate mood symptoms at the time of interview, as assessed by the 24-item Hamilton Rating Scale for Depression^24^ (range 0–24) and Young Mania Rating Scale^25^ (range 0–16); 21% of participants experienced a depressive or manic/hypomanic episode within 1 month before testing, as assessed by the SCID-IV.

### Clinical and cognitive measures

Clinical measures including the number of prior psychotic episodes, suicide attempts, psychiatric hospital admissions and mood episodes were extracted from the SCID-IV. Vulnerability to psychosis, also referred to as schizotypy,^26^ was measured with the Schizotypal Personality Questionnaire (SPQ),^27^ a self-report scale based on the DSM-III-R criteria that includes 74 yes/no questions and can be broken into nine subscales (ideas of reference, suspiciousness, unusual perceptual experiences, no close friends, constricted affect, social anxiety, odd beliefs or magical thinking, odd or eccentric behaviour, odd speech). To assess underlying individual differences in vulnerability to psychosis, we organised the subtypes with the three-factor model:^28^ cognitive–perceptual (ideas of reference, magical thinking, unusual perceptual experiences, odd speech and paranoid ideation), interpersonal (social anxiety, no close friends, constricted affect and paranoid ideation) and disorganised (odd behaviour and odd speech) factors. Higher scores on the SPQ indicate higher vulnerability to psychosis.^29^ We computed scores for each of the three factors of the SPQ (cognitive–perceptual, interpersonal and disorganised) and the total SPQ score for each participant.

Neurocognitive performance was measured with the MATRICS Consensus Cognitive Battery (MCCB),^30^ which uses many individual tests to assess seven domains of cognition: processing speed, attention and vigilance, working memory, verbal learning (we used the California Verbal Learning Test, in place of the Hopkins Verbal Learning Test), visual learning, reasoning and problem solving, and social cognition. All neurocognitive testing data was transformed based on the entire bipolar disorder sample with complete MCCB data (which was reported as normalised T-scores) (*n* = 221) to standardised *z*-score values with a mean of zero and s.d. of one.

We also assessed participants’ level of social functioning using the Social Adjustment Scale Self-Report (SAS-SR),^31^ which examined their functional capacity in regard to paid work, social activities and in family roles.

### Lifetime trauma assessments

Data on the presence of lifetime trauma was extracted primarily from the post-traumatic stress disorder (PTSD) module of the SCID-IV. The PTSD module, also referred to as the trauma module, has been deemed a reliable screening tool.^32^ We used results from the Childhood Trauma Questionnaire (CTQ) to verify some data from the SCID-IV in cases where no trauma module data was available or the personally experienced trauma was acknowledged but the category was not specified (this was done in *n* = 40 participants). We did not cross-check between the PTSD module and CTQ for all participants, to avoid double-counting reported traumas. As the PTSD module includes more direct questions, any affirmative answer on questions L1 or L2 was classified as ‘trauma present’. Questions L3–L5 pertain to witnessing or hearing about a trauma, which is considered to have less of an impact than directly experiencing a traumatic event.^21^ We included traumas in questions L3–L5 only when they were specifically listed with sufficient detail.

The CTQ consists of 28 questions that comprise five subscales: physical abuse, physical neglect, emotional abuse, emotional neglect and sexual abuse. Participants responded on a scale of 1–5, where 1 is ‘never’ and 5 is ‘very often’. The classification of trauma presence from the CTQ was determined by the following scores on questions corresponding to the type of abuse:^33^ ≥10 for physical abuse, ≥10 for physical neglect, ≥13 for emotional abuse, ≥15 emotional neglect and ≥8 for sexual assault.

One author (J.G.L.) abstracted written SCID-IV responses into a spreadsheet. Based on the abstracted responses, classification was completed independently by two members of the research team (J.G.L. and C.E.M.), and discrepancies between raters were reviewed, discussed and resolved. The raters had an initial concordance of approximately 61%. Based on the above-mentioned trauma measures, we re-classified trauma into groups with nomenclature from trauma-informed care models:^21^ naturally occurring, combined interpersonal and accidental, and combined interpersonal and intentional. Naturally occurring traumas are likely unavoidable (e.g. earthquake), whereas interpersonal and accidental traumas and interpersonal and intentional traumas result from human action.

Naturally occurring traumas include tornado, lightning strike, wildfire, avalanche, physical ailment or disease, bereavement, fallen tree, earthquake, dust storm, volcanic eruption, blizzard hurricane, cyclone, typhoon, meteorite flood, tsunami, epidemic, famine, and landslide or fallen boulder. Combined interpersonal and accidental traumas include train derailment, roofing fall, structural collapse, mountaineering accident, aircraft crash, car accident (or other accidents), mine collapse or fire, radiation leak, crane collapse, gas explosion, electrocution, machinery-related accident, oil spill, maritime accident, accidental gun shooting and sports-related death. Combined interpersonal and intentional traumas include arson, terrorism, sexual assault or abuse, homicides or suicides, mob violence or rioting, gang violence, physical abuse or neglect, stabbing or shooting, warfare, domestic violence, poisoned water supply, human trafficking, school violence, torture, home invasion, bank robbery, genocide and medical or food tampering. We added a ‘witness to abuse or violence’ category for those individuals that acknowledged a specific trauma on questions L3–L5 that did not better fit under another category. Participants who had CTQ data included in the grouping analysis were classified as interpersonal and intentional trauma or intentional and accidental trauma.

Three participants reported naturally occurring traumas (one earthquake and two hurricanes). These individuals were added to the accidental trauma group, to create a combination accidental and naturally occurring trauma group. We also created an independent group called the intentional and accidental trauma group for those that experienced both naturally occurring trauma and interpersonal and intentional trauma; each group was independent from the others and no participants were counted twice.

Finally, we calculated a proxy of cumulative trauma burden by summing the number of different trauma types an individual reported based on the SCID-IV (supplemented in some cases by CTQ). Each trauma type (e.g. physical abuse or neglect, fire, mugging) was listed as a separate indicator, even if the trauma(s) occurred multiple times. We were not able to capture or estimate the total number of times a specific trauma occurred over the life course from our data-set.

### Statistical analysis

All analyses were conducted with SPSS Statistics for Windows version 24 (IBM Corporation). To compare statistical differences in demographics between two groups, we used Student's *t*-tests or chi-squared analyses where appropriate. To explore the relationship between type of trauma and schizotypal features on the SPQ, we used a multivariate analysis of covariance (MANCOVA) to establish the mean group differences on the three-factor model of the SPQ, with trauma category (none, naturally occurring, intentional and accidental, interpersonal and intentional) as the fixed factor and age, race (dichotomised), gender, education, depressive and manic symptom severity, and number of psychiatric hospital admissions (as a marker of illness course) as covariates. Then, we assessed the effect of trauma type on cognitive performance using a MANCOVA to assess the seven cognitive domains of the MCCB; we controlled for the same covariates as above. In an exploratory analysis, we used a combination of MANCOVA and analysis of covariance to assess the interaction of gender and a proxy measure for cumulative trauma burden on relevant outcomes. The omnibus tests of our multivariate or univariate analyses were considered significant at a *P*-value of 0.05. Bonferroni correction was applied to statistical tests to control for multiple comparisons, where appropriate (indicated in the Results section).

## Results

### Demographics and clinical characteristics of the sample

Table 1 shows statistical comparisons between those with and without a lifetime history of trauma in our sample. Most participants with bipolar disorder reported a lifetime history of trauma (*n* = 189, 80%). There were no differences in age, education, ethnicity, race, psychosis history, psychotropic medications or DSM diagnosis (bipolar disorder type 1 or 2) between the trauma-negative (*n* = 47) and trauma-positive (*n* = 189) bipolar disorder groups (*P*-values all ≥0.05). The trauma-positive group had more females (55%) relative to males (*χ*^2^ = 4.2, *P* = 0.04), than the trauma-negative group (38% female). The trauma-positive group reported a higher rate of lifetime comorbid anxiety disorders (*χ*^2^ = 4.8, *P* = 0.03), greater number of prior mood episodes (*P* = 0.04) and more psychiatric hospital admissions (*P* = 0.01) compared with the trauma-negative group.

**Table 1:** Characteristics of participants with bipolar disorder with and without a reported history of trauma

	Any trauma, yes/no					
	Trauma negative (*n* = 47)		Trauma positive (*n* = 189)		Test statistic	*P*-value
	Count/mean	%/s.d.	Count/mean	%/s.d.		
Diagnosis						
Bipolar disorder type 1	35	74.5%	157	83.0%	1.8	0.2
Bipolar disorder type 2	12	25.5%	32	17.0%		
Total	47	100%	189	100%		
Gender						
Male	29	62.0%	85	45.0%	4.2	0.04
Female	18	38.0%	104	55.0%		
Total	47	100%	189	100%		
Age, years	41.2	12.9	44.2	12.0	1.5	0.1
Education, years	15.1	2.8	14.5	3.5	1.0	0.3
Race						
Black	14	30.0%	81	42.8%	9.5	0.05
White	28	60.0%	92	48.7%		
Asian	5	10.0%	6	3.2%		
Other	0	0	8	4.2%		
Multi-racial	0	0	2	1.1%		
Total	47	100%	189	100%		
Ethnicity						
Non-Hispanic	39	83.0%	148	78.3%	0.5	0.5
Hispanic	8	17.0%	41	21.7%		
Total	47	100%	189	100%		
Young Mania Rating Scale	2.3	3.3	2.7	3.5	−0.6	0.5
Hamilton Rating Scale for Depression	6.5	6.0	7.4	6.2	−0.9	0.3
Brief Psychiatric Rating Scale (*n* = 235)	21.7	4.3	22.4	4.5	−0.9	0.3
Negative Symptoms Scale (*n* = 234)	5.4	7.8	5.7	7.2	−0.3	0.8
Number of mood episodes (*n* = 228)	18.4	16.4	26.6	25.4	−2.1	0.04
Number of psychiatric hospital admissions	2.3	2.7	5.0	7.0	−2.6	0.01
Number of psychotropic medications (*n* = 235)	1.7	1.3	1.8	1.2	−0.6	0.6
Age at onset, years	22.9	8.7	21.2	7.7	1.3	0.2
Premorbid IQ (Wide Range Acheivement Test-3 Reading) (*n* = 233)	104.3	13.4	103.0	13.8	0.6	0.6
Antidepressants (*n* = 232)						
No	31	67.4%	112	59.3%	0.8	0.4
Yes	15	32.6%	74	40.7%		
Total	46	100%	186	100%		
Lithium (*n* = 232)						
No	36	78.2%	165	88.7%	3.4	0.06
Yes	10	21.8%	21	11.3%		
Total	46	100%	186	100%		
Antipsychotics (*n* = 232)						
No	26	56.5%	89	47.8%	1.1	0.3
Yes	20	43.5%	97	52.2%		
Total	46	100%	186	100%		
Anticonvulsants (*n* = 232)						
No	24	52.2%	102	54.8%	0.1	0.7
Yes	22	47.8%	84	45.2%		
Total	46	100%	186	100%		
Lifetime anxiety disorder (*n* = 233)						
No	29	63.0%	84	44.9%	4.8	0.03
Yes	17	37.0%	103	54.1%		
Total	46	100	187	100		
Lifetime substance use disorder (*n* = 233)						
No	32	70.0%	106	56.7%	2.5	0.1
Yes	14	30.0%	81	43.3%		
Total	46	100%	187	100		
Lifetime psychotic episode						
No	29	61.7%	104	55.0%	0.7	0.4
Yes	18	38.3%	85	45.0%		
Total	47	100%	189	100%		

### Lifetime trauma frequencies

**Fig. 1:**
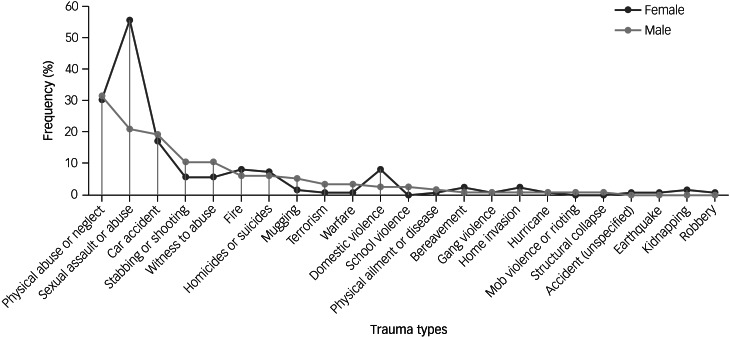
Frequency of reported lifetime traumas in bipolar disorder.

Trauma types abstracted from the SCID-IV trauma module were based on the SAMHSA system of classification (Fig. 1). Of those that reported a lifetime history of trauma, 18 people reported a history of only accidental or naturally occurring trauma. Most of our sample reported a history of only interpersonal and intentional trauma (*n* = 119); 52 participants reported a history of both intentional and accidental trauma. The most frequently reported types of naturally occurring trauma were car accidents (*n* = 43) and fires (*n* = 17). The most frequently reported types of interpersonal and intentional trauma were physical abuse or physical neglect (*n* = 73), and sexual assault or abuse (*n* = 92). Other common types of interpersonal and intentional trauma included being a witness to abuse (*n* = 19), stabbings or shootings (*n* = 19) and homicides or suicides (*n* = 16).

### The relationship between lifetime trauma categorisation and schizotypal features and cognition

**Fig. 2:**
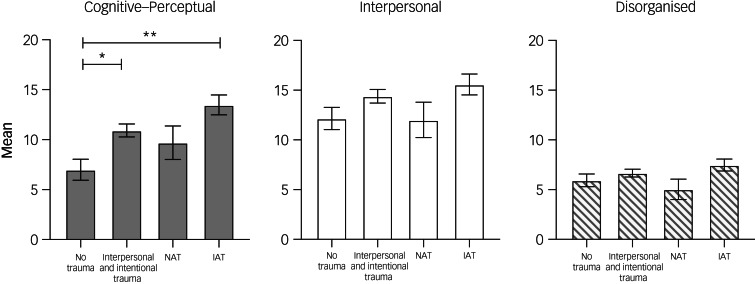
The effect of trauma type on schizotypal features in participants with bipolar disorder. Shown here are estimated marginal means and s.e. from a multivariate analysis of covariance, with Schizotypal Personality Questionnaire domains as dependent variables and trauma group as the fixed factor. Significance: *P < 0.05, **P < 0.01. No trauma (*n* = 47); naturally occurring and accidental trauma (NAT; *n* = 18); interpersonal and intentional trauma (*n* = 119); intentional and accidental trauma (IAT; *n* = 52).

Results of a MANCOVA (controlling for gender, age, race (dichotomised), education, prior psychiatric hospital admissions and current mood symptom severity) are shown in Fig. 2. We found a significant overall effect of trauma type on the SPQ (Wilks’ *λ*: F(9542.9) = 2.6, *P* = 0.005). Individual tests showed a significant effect of trauma type on the cognitive–perceptual domain (F(3) = 6.7, *P* < 0.001). Pairwise comparisons (Bonferroni correction applied) between groups showed that no trauma was significantly different from accidental and intentional trauma (*P* < 0.001) and interpersonal and intentional trauma (*P* = 0.01). We also found a significant main effect of gender on the SPQ (F(3223) = 4.2, *P* = 0.007). In our second main analysis (same covariates as above) examining cognition from the MCCB (*n* = 221), we did not find a significant main effect of trauma group on cognitive performance (F(21 586.3) = 0.9, *P* = 0.5).

### The cumulative burden of trauma: effects on clinical features of illness in males and females with bipolar disorder

Based on our result of a significant main effect of gender on SPQ (reported above), as well as the robust body of literature indicating gender-specific differences in response to trauma on mood and psychotic symptoms,^11^ cognitive performance^34^ and function in patients, we focused on gender effects in the subsequent exploratory analyses related to cumulative trauma burden. Figure 1 shows the frequency of reported trauma types in males and females. In females, the most frequently reported trauma types were sexual assault or abuse (55%), physical abuse or neglect (30%) and car accidents (17%). Similarly, males most reported physical abuse or neglect (32%), sexual assault or abuse (21%) and car accidents (19%)**.**

We estimated the cumulative burden of lifetime trauma by summing the total number of reported trauma types in our bipolar disorder sample; we defined ‘high trauma burden’ as having reported three or more different traumas, and ‘low trauma burden’ as fewer than three different traumas. High (*n* = 34) and low (*n* = 202) trauma burden groups did not differ by current depressive symptoms, education, age, gender, ethnicity, race (trend level), psychosis, bipolar diagnosis (type 1 or 2), psychotropic medications (yes/no), lifetime comorbid substance misuse, lifetime comorbid anxiety disorder (trend level), current cannabis use and current alcohol use (all *P-*values ≥0.05). There was a difference between groups on current manic symptoms (higher in the high trauma burden group, *P* = 0.049) and lifetime manic episodes (higher in the high trauma burden group, *P* = 0.04). We performed exploratory analyses examining the interaction of gender and trauma burden on vulnerability to psychosis, cognition and social function.

#### Vulnerability to psychosis

In a multivariate analysis (controlled for age, race (dichotomised), number of psychiatric hospital admissions, education and current mood symptoms) with dependent variables including the three subscores of the SPQ (disorganised, interpersonal and cognitive–perceptual), we did not find a significant interaction between gender and trauma burden (high versus low) (F(3224) = 0.4, *P* = 0.7).

#### Cognition

In a multivariate analysis (controlled for age, race (dichotomised), number of psychiatric hospital admissions, education and mood symptoms) with dependent variables including the seven domains of the MCCB (speed of processing, attention and vigilance, working memory, visual learning, verbal learning, reasoning and problem solving, and social cognition), we found a non-significant interaction between gender and trauma burden (F(7205) = 1.3, *P* = 0.2).

#### Social function

In a multivariate analysis (controlled for same as above) with dependent variables of social leisure and family outside home on the SAS-SR, we did not find a significant interaction between gender and trauma burden (F(2220) = 2.0, *P* = 0.13).

## Discussion

We assessed the relationship between lifetime trauma and clinical outcomes, vulnerability to psychosis, cognition and social function in 236 participants with bipolar disorder. We found that most participants with bipolar disorder have a lifetime history of trauma and most reported only interpersonal and intentional traumas (*n* = 119). Physical abuse and/or neglect and sexual abuse and/or abuse were the two most common types of interpersonal and intentional trauma reported, and car accidents were the most common types of naturally occurring or accidental trauma reported. Our main findings indicate that trauma type and cumulative burden of trauma are associated with increased schizotypal features and a poorer clinical course of bipolar disorder.

Participants who reported interpersonal and intentional trauma or intentional and accidental trauma showed increased schizotypal symptoms compared with participants with no trauma, marked by higher scores on the SPQ (Fig. 2). Specifically, our analysis showed that those with a lifetime history of interpersonal and intentional trauma or intentional and accidental trauma scored higher on the cognitive–perceptual (e.g. ideas of reference, magical thinking, unusual perceptual experiences, odd speech and paranoid ideation) subscale of the SPQ compared with participants with no trauma. Notably, we did not find a significant main effect of trauma on presence of lifetime threshold psychotic symptoms in our bipolar disorder sample (e.g. bipolar disorder psychotic diagnostic subtype), which suggests that the SPQ may be capturing more subtle and persistent aspects of the vulnerability to psychosis spectrum than the DSM is able to define in the context of a diagnosis of bipolar disorder. The diagnosis of threshold level psychosis in bipolar disorder is a difficult one to make with certainty because of the presence of acute manic and/or depressive symptoms and the transient nature of the psychotic symptoms in bipolar disorder. Overall, these results confirm previous findings^6,35^ that strongly indicate that a history of interpersonal and intentional trauma is related to the heightened presence of both positive (e.g. hallucinations, delusions) and negative (e.g. avolition, alogia) symptoms of psychosis regardless of diagnosis (schizophrenia, bipolar disorder or healthy control).

Further, the naturally occurring trauma group generally scored lower on the SPQ scales than the interpersonal and intentional trauma and intentional and accidental trauma groups (although not significant). This result is in line with a report by Ford et al that examined healthy women with and without a trauma history, and found that those in a ‘single incident of non-interpersonal trauma (SNI)’ group reported less affective dysregulation and fewer dissociative features compared with a ‘cumulative abuse trauma’ group.^36^ In this study, the SNI group performed similarly to the group with no trauma history, which we also found in our study.^36^

Understanding the aetiological link between trauma and psychosis/vulnerability to psychosis is important as over half of people with bipolar disorder are estimated to experience psychotic symptoms in their lifetime (including delusions and hallucinations), and the presence of psychosis is associated with a more burdensome clinical course, including more frequent hospital admissions and earlier onset of illness.^37^ Although the root of the relationship between trauma and vulnerability to psychosis has not been fully elucidated, psychotic symptoms can be triggered by trauma even in otherwise healthy people, with no diagnosis of a major mood or psychotic disorder.^6^ It is thought that trauma may create a biological^38^ or psychological vulnerability to the development of psychotic features.^39^

Various hypotheses exist to explain this well-established link: one hypothesis suggests that trauma causes affective dysregulation, which then increases the risk of psychosis.^40^ Others propose that the effect of dissociation during trauma may lead to further vulnerability to ‘breaks with reality’ such as hallucinations.^39^ Moreover, trauma may negatively alter core beliefs, making an individual more likely to view the world negatively and become susceptible to experience delusions such as persecution.^40^ The fact that individuals who experience interpersonal and intentional traumas may also experience an increase in psycho-somatosensory activation and fragmentation of events^41^ could help explain why our sample scored higher on the cognitive–perceptual dimension of the SPQ.

Biological models suggest that trauma may alter feedback properties of the hypothalamic-pituitary-adrenal axis as well as reduce hippocampal volume.^42^ Animal studies have shed some light on the link between extreme stress and brain function; one study found that severely aversive social stressors (being exposed to a dominating conspecific) can increase dopamine transmission in the mesolimbic pathway of the brain, which has been found to be affected in individuals with positive symptoms of psychosis.^43^

Our study also examined the interaction between cumulative trauma burden and gender on clinical and cognitive features (SPQ, cognition and social functioning) in bipolar disorder. Our results were largely negative, with a few trend-level results. Prior studies have found gender-specific differences in the effect of trauma on cognition in bipolar disorder, although the literature is mixed. In one study, childhood abuse (particularly emotional and physical neglect) was associated with poorer social cognition in both genders.^44^ Similarly, we previously reported gender-specific effects of emotional abuse on several measures of affective processing in patients with bipolar disorder.^20^ In another study, the presence of trauma in males with psychosis was significantly correlated with impaired performance across multiple cognitive domains, compared with males with no reported trauma.^16^

Our study is consistent with prior work that suggests that participants with mood disorders are exposed to multiple traumas over their life course.^10^ Although many studies have demonstrated the dose–response effect of childhood adverse experiences on clinical outcomes in bipolar disorder, few studies have found a correlation between number of lifetime traumas and clinical outcomes. One study demonstrates that the presence of childhood adversity in combination with negative lifetime socioeconomic factors have a synergistic effect in increasing risk for psychosis.^45^ In an exploratory analysis, we did not find an interaction between trauma and race on our primary outcome measures (data not shown). Our findings suggest that experiencing multiple lifetime types of traumas may exacerbate the clinical course of bipolar disorder and lead to poorer outcomes. These findings also highlight the need for trauma inquiry^46^ in patients with bipolar disorder, and consideration of how trauma-focused or trauma-informed treatments may be integrated into treatment planning to improve outcomes.

There are limitations to our study. First, although our overall sample was large (*N* = 236), the subgroup of people who did not report trauma was relatively small (*n* = 47), although this is consistent with prior reports of an unfortunately high rate of trauma in bipolar disorder. Further, the naturally occurring trauma group (*n* = 18) and intentional and accidental trauma group (*n* = 52) were much smaller than the interpersonal and intentional trauma group (*n* = 119), which could affect the strength of our conclusions. One possible explanation for the limited number of naturally occurring traumas reported is the assessments used (e.g. the SCID-IV PTSD module and the CTQ focus largely on interpersonal and intentional sources of trauma); therefore, future studies should ensure that trauma assessments include queries for ‘non-interpersonal’ type traumas.

Another limitation is the retrospective nature of collecting our trauma data; both the SCID-IV and CTQ are retrospective measures that rely on self-reports, and we did not independently verify any information with family members or past physicians, but instead relied solely on the participants’ reports. In some cases, participants did not explicitly report sexual or physical assault during the SCID-IV, but did endorse it on the CTQ. This may be because of participants’ reluctance to openly discuss prior traumas, as the SCID-IV is a clinician-based interview, whereas, they may have been more inclined to note this history on a self-report. We did not explicitly ask the participant to indicate the timing of each of the traumas that we analysed. We generally inferred this information based on participants’ responses.

Our cumulative trauma burden measure is limited as it does not quantify the total number of traumas. Further, there is potential bias in our sample as most participants were recruited from two institutions, highly educated and symptomatically stable (based on the CGI-BD) at the time of the interview. Although higher education may imply less trauma exposure, most participants in our sample were exposed to trauma.

In conclusion, our data suggest that the cumulative burden of trauma and the type of trauma may contribute to the severity of illness of bipolar disorder. In our sample, 80% of participants (*n* = 189) reported at least one trauma and 14.4% (*n* = 34) participants reported experiencing three or more distinct types of traumas. Our study is consistent with prior studies that report high rates of trauma in bipolar disorder. Importantly, relying solely on only the CTQ as a trauma measure would have excluded participants that experienced trauma in adulthood. Our findings suggest that trauma may not only influence the onset of bipolar disorder, but also exacerbate the clinical course, as cumulative trauma burden was associated with several negative clinical outcomes (higher manic symptoms and lifetime manic episodes). This is in line with the broader literature suggesting that ACEs are a risk factor for poorer health outcomes,^47^ as well as onset of bipolar disorder.^48^ Lifetime trauma is certainly not the only factor affecting clinical course in bipolar disorder; however, our results indicate the significant impact that lifetime trauma may have on psychotic features and cognitive dysfunction in bipolar disorder. These findings also support a role for trauma inquiry in risk stratification and treatment planning for patients with bipolar disorder.

## Data Availability

The data that support the findings of this study are available from the corresponding author, K.E.B., upon reasonable request.
